# The effect of change in fasting glucose on the risk of myocardial infarction, stroke, and all-cause mortality: a nationwide cohort study

**DOI:** 10.1186/s12933-018-0694-z

**Published:** 2018-04-07

**Authors:** Gyeongsil Lee, Sung Min Kim, Seulggie Choi, Kyuwoong Kim, Su-Min Jeong, Joung Sik Son, Jae-Moon Yun, Sang Min Park

**Affiliations:** 1 Department of Family Medicine, Health Promotion Center, Chung-Ang University Hospital, Seoul, South Korea; 2 Clinical Medical Sciences, College of Medicine, Seoul National University, Seoul, South Korea; 3 Department of Family Medicine, Seoul National University Hospital, Seoul, South Korea

**Keywords:** Prediabetes, Blood glucose, Myocardial infarction, Stroke, Mortality

## Abstract

**Background:**

The effect of change in blood glucose levels on the risk of cardiovascular disease among individuals without diabetes is currently unclear. We aimed to examine the association of change in fasting serum glucose with incident cardiovascular disease and all-cause mortality among representative large population.

**Methods:**

We analyzed the data from retrospective cohort of Korean National Health Insurance Service. In total, 260,487 Korean adults aged over 40 years, without diabetes mellitus and cardiovascular disease at baseline measured change in fasting serum glucose according to the criteria of impaired and diabetic fasting glucose status: normal fasting glucose (NFG, fasting glucose: < 100 mg/dL), impaired fasting glucose (IFG, fasting glucose: 100.0–125.9 mg/dL), and diabetic fasting glucose (DFG, fasting glucose: ≥ 126.0 mg/dL). Compared to the persistently unchanged group (i.e. NFG to NFG or IFG to IFG), Cox proportional hazards regression analyses were performed in the changed group to obtain the hazards ratio (HR) with 95% confidence interval (CI) for the subsequent median 8-year myocardial infarction, stroke, and all-cause mortality.

**Results:**

Compared to individuals with persistent NFG (i.e., NFG to NFG), individuals who shifted from NFG to DFG had an increased risk of stroke (HR [95% CI]: 1.19 [1.02–1.38]) and individuals who shifted from NFG to IFG or DFG had increased risks of all-cause mortality (HR [95% CI]: 1.08 [1.02–1.14] for NFG to IFG and 1.56 [1.39–1.75] for NFG to DFG). Compared to individuals with persistent IFG, individuals who shifted from IFG to DFG had an increased risk of MI and all-cause mortality (HR [95% CI]: 1.65 [1.20–2.27] and 1.16 [1.02–1.33], respectively).

**Conclusions:**

Increasing fasting glucose in non-diabetic population is associated with risks of the MI, stroke, and all-cause mortality, which is more rapid, more severe.

**Electronic supplementary material:**

The online version of this article (10.1186/s12933-018-0694-z) contains supplementary material, which is available to authorized users.

## Background

Diabetes is related to both microvascular and macrovascular complications as well as mortality [1, 2]. In particular, macrovascular complications such as myocardial infarction (MI) and stroke account for 80% of all deaths in patients with type 2 diabetes mellitus [3]. Therefore, preventing macrovascular complications by controlling hyperglycemia is imperative in reducing the risk of cardiovascular disease and mortality [4–6].

Recent evidence suggests that a prediabetic status may also elevate the risk of cardiovascular disease and mortality. A prediabetic status referred to people who have impaired fasting glucose (IFG, fasting glucose of 100–125 mg/dL), impaired glucose tolerance (IGT, 2-h post glucose load of 140–199 mg/dL), or both, according to American Diabetes Association [5]. Some studies have shown that IGT is superior to IFG as a risk factor for cardiovascular disease [7–10]. However, the Australian Diabetes, Obesity, and Lifestyle Study (AusDiab) reported IFG as well as IGT to be an independent predictor of cardiovascular disease and all-cause mortality [11]. Moreover, meta-analyses also showed that not only IGT but IFG is related to risk of cardiovascular disease [12, 13].

Blood glucose levels in most prior studies, however, were assessed only one time, possibly resulting in misclassification of IFG or IGT. The effect of change in blood glucose levels on the risk of cardiovascular disease among people without diabetes are currently unclear. We, therefore, aimed to examine the association of change in fasting serum glucose with incident cardiovascular disease and all-cause mortality within a large population without diabetes using the Korean National Health Insurance Service-Nation Health Screening Cohort (NHIS-HEALS).

## Methods

### Study population

The study population was derived from the NHIS-HEALS database from January 1, 2002 to December 31, 2013, which is provided by the NHIS (NHIS-2017-2-460). In 2000, NHIS was launched by integrating diverse medical insurance organizations in Korea. The NHIS has provided mandatory health insurance for all Koreans since 1989; thus, the enrollment rate is nearly 98% [14]. Attrition over follow-up in this database is known to be rare because the NHIS acts as a universal health insurance [15]. Enrollees over 40 years of age are required to take bi-annual health screening visits, including gathering of clinical and sociodemographic data. Among the entire data set from the national health examination, 10% (about 500,000 participants) of the data are collected by simple random sampling with deidentification [15]. NHIS-HEALS contains retrospective data, including electronic medical records, information on clinical visits, diagnosis based on International Classification of Disease (ICD) codes, anthropometry, and laboratory examination. Sociodemographic data on age, sex, income status, residential information, and disability status are also included. The NHIS plays a role in paying premiums to medical institutions. For these reasons, data are known to be accurate [15].

A total of 334,377 participants who took at least two visits of health screening between the first (from 2002 to 2003) and second (from 2004 to 2005) health examinations, with fasting serum glucose values, were selected. Among these individuals, we excluded 42,097 participants who were diagnosed with type 2 diabetes before the onset of follow-up (index date: January 1, 2006) according to the ICD-10 codes (10th revision) by the World Health Organization for type 2 diabetes (E11, E12, E14) or those with baseline fasting glucose levels ≥ 126.0 mg/dL at their first health examination. We further excluded 780 participants who passed away and 30,753 participants who were diagnosed MI or stroke before the index date. Then, we removed 122 participants with fasting glucose < 50.0 mg/dL and 138 participants without sex values. Finally, the study population consisted of 260,487 participants (149,913 men, 110,574 women).

### Data collection

**Fig. 1:**
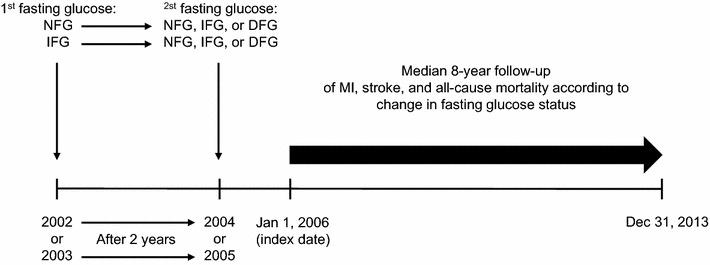
Timeline of the study design. Subjects without diabetes and cardiovascular disease performed 2-year fasting serum glucose examination. The first fasting glucose status was categorized into two groups, normal fasting glucose [(NFG), fasting serum glucose: < 100.0 mg/dL] and impaired fasting glucose [(IFG), fasting serum glucose: 100.0–125.9 mg/dL] (individuals with more than 126 mg/dL were excluded). The second fasting glucose status was categorized into three groups, NFG, IFG, and diabetic fasting glucose [(DFG), fasting serum glucose: ≥ 126.0 mg/dL]. Accordingly, six categories based on the change in fasting glucose level were followed up during 8 years for determining the risk of myocardial infarction (MI), stroke, and all-cause mortality

The participants underwent blood examination including fasting glucose levels during each health visit. We measured changes in fasting serum glucose levels from the first health examination to the second health examination in this study (Fig. 1). Fasting glucose levels of the first health examination were divided into two groups: normal fasting glucose (NFG, fasting glucose: < 100 mg/dL) and impaired fasting glucose (IFG, fasting glucose: 100.0–125.9 mg/dL). Fasting glucose levels of the second health examination were divided into 3 groups: NFG, IFG, and diabetic fasting glucose (DFG, fasting glucose: ≥ 126.0 mg/dL). The changes in fasting glucose were defined as the shift from each fasting glucose level of the first health examination (baseline) to each fasting glucose of the second health examination after 2 years.

The main outcomes of this study were hospitalizations due to MI or stroke, and all-cause mortality that occurred from January 1, 2006 and December 31, 2013. ICD-10 codes were used to identify and classify the outcomes: MI (I21–I24), stroke (I60–69). Hospitalization due to cardiovascular events was defined as participants who were hospitalized for 2 days or more due to the relevant disease, in order to exclude admissions for other diseases.

Covariates were based on the data from near before index year included age, sex, socioeconomic status (low and high), body mass index (BMI, kg/m^2^), smoking status (never and ever), alcohol consumption (none, < 3, and ≥ 3 times per week), physical activity (no, < 3, and ≥ 3 times per week), systolic/diastolic blood pressure (mmHg), total cholesterol (mg/dL), Charlson Comorbidity Index (CCI, 0, 1–2, and ≥ 3). Participants were classified as underweight, normal weight, overweight, and obese based on the Asian criteria [16]. CCI is the most commonly used comorbidity index for predicting mortality [17, 18].

### Statistical analysis

Cox proportional hazards regression analyses were performed to obtain the hazards ratio (HR) with 95% confidence interval (CI) of MI, stroke, and all-cause mortality for each NFG and IFG group at the first health examination, after adjusting for age, sex, socioeconomic status, BMI, smoking status, alcohol consumption, physical activity, CCI, blood pressure, total cholesterol, and baseline fasting glucose level. Among covariates, age, BMI, blood pressure, total cholesterol and fasting glucose were dealt with continuous variables. Those with persistently unchanged fasting glucose (NFG at the first health examination to NFG at the second health examination and IFG at the first health examination to IFG at the second health examination) were considered the reference group. Stratified analyses were conducted including age, sex, and BMI in order to identify potential subgroups that show a significant association between change in fasting glucose and MI, stroke, and all-cause mortality. After excluding participants who were diagnosed with myocardial infarction or stroke, or who died between January 1, 2006 and December 31, 2006, and between January 1, 2006 and December 31, 2007, sensitivity analyses were performed to enhance the reliability of the association between change in fasting glucose levels and MI, stroke, and mortality. All data mining and statistical analyses in this study were conducted using SAS 9.4 (SAS Institute, Cary, NC, USA) and STATA 13.0 (StataCorp LP, College Station, TX, USA). Statistical significance of this study was defined as a two-sided *p* value less than 0.05.

## Results

260,487 individuals without diabetes were followed up to an average of 8 years (standard deviation, SD 0.9), resulting in 2,042,960 person-years. During the follow-up, the number of incident MI, incident stroke, and all-cause mortalities were 1318 (0.5%), 8144 (3.13%), 10,065 (3.86%), respectively. The baseline characteristics of study participants are presented in Table 1. Those who belonged to the NFG group at the baseline were more likely to be younger women, with a lower BMI, never smokers, with a lower alcohol consumption, lower blood pressure, and lower total cholesterol than those who were in the IFG group at baseline.

**Table 1:** Baseline characteristics of study participants

	Fasting glucose (mg/dL)		
	NFG (< 100.0 mg/dL)	IFG (100.0–125.9 mg/dL)	*p* value
Number of subjects, n	198,560	61,927	
Age, years, mean (SD)	53.5 (8.6)	54.7 (8.9)	
40–49, %	41.6	35.8	< 0.001
50–59, %	34.8	35.9	
60–69, %	17.5	20.9	
≥ 70, %	6.1	7.4	
Sex, %			
Men	55.3	64.6	
Women	44.7	35.4	
Socioeconomic status, %			
Low	34.2	35.4	< 0.001
High	65.5	64.2	
BMI, kg/m^2^, mean (SD)	23.7 (2.8)	24.2 (2.9)	
<18.5, %	2.5	1.9	< 0.001
18.5-22.9, %	39.2	32.8	
23-24.9, %	27.8	28.3	
≥25, %	30.5	37.0	
Smoking status, %			
Never smoker	66.2	61.4	< 0.001
Ever smoker	29.8	34.6	
Alcohol consumption, per week, %			
< 3 times	88.9	84.9	< 0.001
≥ 3 times	9.5	13.6	
Physical activity, per week, %			
< 3 times	76.2	75.9	0.092
≥ 3 times	21.6	22.0	
CCI, %			
0	36.5	36.8	< 0.001
1–2	50.9	49.5	
≥ 3	12.6	13.7	
Systolic BP, mmHg, mean (SD)	124.6 (16.7)	128.1 (17.1)	< 0.001
Diastolic BP, mmHg, mean (SD)	78.3 (11.0)	80.3 (11.1)	< 0.001
Total cholesterol, mg/dL, mean (SD)	197.3 (36.0)	200.1 (37.1)	<0.001

*SD* standard deviation, *NGT* normal glucose tolerance, *IFG* Impaired fasting glucose, *BMI* body mass index, *CCI* Charlson comorbidity index, *BP* blood pressure

Participants were classified as underweight, normal weight, overweight, and obese based on the Asian criteria

**Fig. 2:**
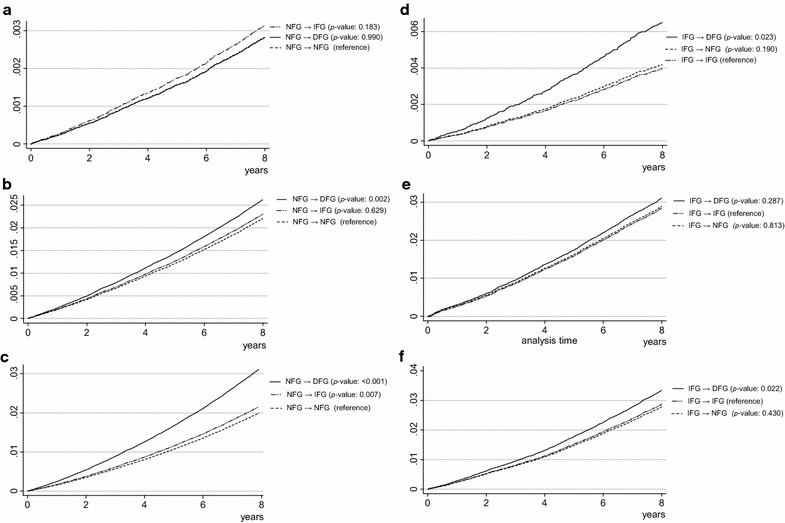
Adjusted cumulative hazard curves for 8-year MI, stroke, and all-cause mortality by change in fasting glucose. Hazard ration analyzed by Cox proportional hazards regression analysis adjusted for age, sex, socioeconomic status, physical activity, smoking status, alcohol consumption, body mass index, blood pressure, total cholesterol, Charlson comorbidity index, and baseline fasting glucose level. *MI* myocardial infarction, *NFG* normal fasting glucose, *IFG* impaired fasting glucose, *DFG* diabetic fasting glucose.** a** Cumulative hazard of MI among individuals with NFG at the 1st examination.** b** Cumulative hazard of stroke among individuals with NFG at the 1st examination.** c** Cumulative hazard of all-cause mortality among individuals with NFG at the 1st examination.** d** Cumulative hazard of MI among individuals with IFG at the 1st examination.** e** Cumulative hazard of stroke among individuals with IFG at the 1st examination.** f** Cumulative hazard of all-cause mortality among individuals with IFG at the 1st examination

Figure 2 shows adjusted cumulative hazard curves for 8-year MI, stroke, and all-cause mortality by changes in fasting glucose, after adjusting for age, socioeconomic status, smoking habit, alcohol consumption, physical activity, BMI, CCI, blood pressure, and fasting glucose at baseline. Compared to individuals with persistent NFG (i.e., NFG to NFG), individuals who shifted from NFG to DFG had an increased risk of stroke (HR [95% CI]: 1.19 [1.02–1.38]) and individuals who shifted from NFG to IFG or DFG had increased risks of all-cause mortality (HR [95% CI]: 1.08 [1.02–1.14] for NFG to IFG and 1.56 [1.39–1.75] for NFG to DFG). Compared to individuals with persistent IFG, individuals who shifted from IFG to DFG had an increased risk of MI and all-cause mortality (HR [95% CI]: 1.65 [1.20–2.27] and 1.16 [1.02–1.33], respectively).

Subgroup analyses using the multivariable model of MI, stroke, and all-cause mortality risks by changes in fasting glucose from NFG and IFG are shown in Tables 2, 3, 4. Individuals who were 65 years or older had an increased risk for MI. With regard to all-cause mortality, results of all subgroups stratified by age, sex, and BMI were similar to the main results. The magnitude of HRs of all-cause mortality was higher in patients who were more than 50 years old than in those less than 50 years, in men than in women, and those with BMI ≥ 25 kg/m^2^ (obese based on Asian criteria) than with BMI < 25 kg/m^2^.

The results of the sensitivity analyses based on the multivariable model of MI, stroke, and all-cause mortality risks, in relation to changes in fasting glucose, from NFG and IFG, are shown in Additional file 1: Tables S1 and S2. The results were almost similar to the results shown in Fig. 2.

**Table 2:** Subgroup analysis of the associations between change in fasting glucose and the risk of myocardial infarction

Fasting glucose level at baseline (mg/dL)	NFG (< 100.0 mg/dL)			IFG (100.0–125.9 mg/dL)		
Fasting glucose level after 2-year (mg/dL)	NFG (< 100.0 mg/dL)	IFG (100.0–125.9 mg/dL)	DFG (≥ 126.0 mg/dL)	NFG (< 100.0 mg/dL)	IFG (100.0–125.9 mg/dL)	DFG (≥ 126.0 mg/dL)
Age < 65 years						
Subtotal (N)	136,764	32,057	3188	28,272	19,977	3615
Number of cases (n)	482	151	15	124	92	31
HR (95% CI)	1	1.06 (0.88–1.28)	0.85 (0.51–1.42)	1.15 (0.88–1.51)	1	1.49 (0.98–2.25)
Age ≥ 65 years						
Subtotal (N)	19,957	5765	829	5335	3879	849
Number of cases (n)	199	74	12	65	51	22
HR (95% CI)	1	1.23 (0.94–1.61)	1.26 (0.69–2.32)	0.93 (0.64–1.34)	1	2.02 (1.21–3.35)
Men						
Subtotal (N)	83,348	23,763	2790	20,418	16,354	3240
Number of cases (n)	539	183	20	147	120	43
HR (95% CI)	1	1.10 (0.93–1.31)	0.92 (0.58–1.43)	1.04 (0.81–1.32)	1	1.57 (1.10–2.23)
Women						
Subtotal (N)	73,373	14,059	1227	13,189	7502	1224
Number of cases (n)	142	42	7	42	23	10
HR (95% CI)	1	1.11 (0.78–1.57)	1.25 (0.55–2.85)	1.16 (0.69–1.95)	1	2.11 (0.99–4.47)
BMI < 25.0 kg/m^2^						
Subtotal (N)	111,438	24,195	2411	22,436	14,195	2368
Number of cases (n)	439	138	15	112	81	27
HR (95% CI)	1	1.14 (0.94–1.38)	0.91 (0.54–1.56)	0.98 (0.74–1.31)	1	1.71 (1.10–2.65)
BMI ≥ 25.0 kg/m^2^						
Subtotal (N)	45,255	13,615	1605	11,157	9651	2095
Number of cases (n)	242	87	12	77	62	26
HR (95% CI)	1	1.05 (0.82–1.35)	1.10 (0.62–1.98)	1.19 (0.85–1.67)	1	1.63 (1.02–2.60)

Hazard ratio analyzed by Cox proportional hazards regression analysis adjusted for age, sex, socioeconomic status, physical activity, smoking status, alcohol consumption, body mass index, blood pressure, total cholesterol, Charlson comorbidity index, and baseline fasting glucose level

*n* number, *HR* hazard ratio, *CI* confidential interval, *NFG* normal fasting glucose (< 100.0 mg/dL), *IFG* impaired fasting glucose (100.0–125.9 mg/dL), *DFG* diabetic fasting glucose (≥ 126.0 mg/dL)

**Table 3:** Subgroup analysis of the associations between change in fasting glucose and the risk of stroke

Fasting glucose level at baseline (mg/dL)	NFG (< 100.0 mg/dL)			IFG (100.0–125.9 mg/dL)		
Fasting glucose level after 2-year (mg/dL)	NFG (< 100.0 mg/dL)	IFG (100.0–125.9 mg/dL)	DFG (≥ 126.0 mg/dL)	NFG (< 100.0 mg/dL)	IFG (100.0–125.9 mg/dL)	DFG (≥ 126.0 mg/dL)
Age < 65 years						
Subtotal (N)	136,764	32,057	3188	28,272	19,977	3615
Number of cases (n)	2687	747	95	703	518	107
HR (95% CI)	1	1.06 (0.97–1.15)	1.19 (0.97–1.47)	1.03 (0.91–1.15)	1	1.01 (0.82–1.24)
Age ≥ 65 years						
Subtotal (N)	19,957	5765	829	5335	3879	849
Number of cases (n)	1726	527	87	479	366	102
HR (95% CI)	1	1.04 (0.95–1.15)	1.20 (0.97–1.50)	0.99 (0.87–1.14)	1	1.22 (0.98–1.53)
Men						
Subtotal (N)	83,348	23,763	2790	20,418	16,354	3240
Number of cases (n)	2416	790	117	717	599	149
HR (95% CI)	1	1.05 (0.97–1.14)	1.16 (0.96–1.40)	1.00 (0.90–1.12)	1	1.08 (0.90–1.30)
Women						
Subtotal (N)	73,373	14,059	1227	13,189	7502	1224
Number of cases (n)	1997	484	65	465	285	60
HR (95% CI)	1	1.03 (0.94–1.14)	1.25 (0.97–1.60)	1.03 (0.89–1.20)	1	1.08 (0.82–1.43)
BMI < 25.0 kg/m^2^						
Subtotal (N)	111,438	24,195	2411	22,436	14,195	2368
Number of cases (n)	3030	819	113	756	534	114
HR (95% CI)	1	1.06 (0.98–1.15)	1.18 (0.97–1.42)	0.97 (0.87–1.09)	1	1.07 (0.88–1.32)
BMI ≥ 25.0 kg/m^2^						
Subtotal (N)	45,255	13,615	1605	11,157	9651	2095
Number of cases (n)	1383	455	69	426	349	95
HR (95% CI)	1	1.00 (0.90–1.12)	1.20 (0.94–1.53)	1.09 (0.94–1.26)	1	1.12 (0.89–1.41)

Hazard ratio analyzed by Cox proportional hazards regression analysis adjusted for age, sex, socioeconomic status, physical activity, smoking status, alcohol consumption, body mass index, blood pressure, total cholesterol, Charlson comorbidity index, and baseline fasting glucose level

*n* number, *HR* hazard ratio, *CI* confidential interval, *NFG* normal fasting glucose (< 100.0 mg/dL), *IFG* impaired fasting glucose (100.0–125.9 mg/dL), *DFG* diabetic fasting glucose (≥ 126.0 mg/dL)

**Table 4:** Subgroup analysis of the associations between change in fasting glucose and the risk of all-cause morality

Fasting glucose level at baseline (mg/dL)	NFG (< 100.0 mg/dL)			IFG (100.0–125.9 mg/dL)		
Fasting glucose level after 2-year (mg/dL)	NFG (< 100.0 mg/dL)	IFG (100.0–125.9 mg/dL)	DFG (≥ 126.0 mg/dL)	NFG (< 100.0 mg/dL)	IFG (100.0–125.9 mg/dL)	DFG (≥ 126.0 mg/dL)
Age < 65 years						
Subtotal (N)	136,764	32,057	3188	28,272	19,977	3615
Number of cases (n)	2,5864	746	146	700	541	137
HR (95% CI)	1	1.07 (0.98–1.16)	1.72 (1.46–2.04)	0.94 (0.84–1.05)	1	1.23 (1.02–1.48)
Age ≥ 65 years						
Subtotal (N)	19,957	5765	829	5335	3879	849
Number of cases (n)	2661	855	168	816	579	152
HR (95% CI)	1	1.11 (1.03–1.20)	1.46 (1.25–1.71)	1.01 (0.91–1.13)	1	1.17 (0.98–1.40)
Men						
Subtotal (N)	83,348	23,763	2790	20,418	16,354	3240
Number of cases (n)	3726	1213	252	1123	842	214
HR (95% CI)	1	1.07 (1.01–1.14)	1.60 (1.40–1.82)	1.01 (0.92–1.11)	1	1.12 (0.96–1.30)
Women						
Subtotal (N)	73,373	14,059	1227	13,189	7502	1224
Number of cases (n)	1499	388	62	393	278	75
HR (95% CI)	1	1.09 (0.97–1.22)	1.36 (1.06–1.76)	0.87 (0.75–1.02)	1	1.29 (0.99–1.67)
BMI < 25.0 kg/m^2^						
Subtotal (N)	111,438	24,195	2411	22,436	14,195	2368
Number of cases (n)	3983	1188	224	1160	777	193
HR (95% CI)	1	1.11 (1.04–1.18)	1.50 (1.31–1.72)	1.00 (0.92–1.10)	1	1.21 (1.03–1.42)
BMI ≥ 25.0 kg/m^2^						
Subtotal (N)	45,255	13,615	1605	11,157	9651	2095
Number of cases (n)	1241	412	90	355	343	96
HR (95% CI)	1	1.00 (0.89–1.12)	1.65 (1.33–2.04)	0.92 (0.79–1.07)	1	1.08 (0.86–1.36)

Hazard ratio analyzed by Cox proportional hazards regression analysis adjusted for age, sex, socioeconomic status, physical activity, smoking status, alcohol consumption, body mass index, blood pressure, total cholesterol, Charlson comorbidity index, and baseline fasting glucose level

*n* number, *HR* hazard ratio, *CI* confidential interval, *NFG* normal fasting glucose (< 100.0 mg/dL), *IFG* impaired fasting glucose (100.0–125.9 mg/dL), *DFG*, diabetic fasting glucose (≥ 126.0 mg/dL)

## Discussion

In this general population-based, retrospective cohort study with more than 2,000,000 person-years of follow-up, we examined the association between 2-year change in fasting serum glucose and risk of cardiovascular disease and all-cause mortality after a median follow-up of 8 years, after adjusting for cardiovascular covariates. Among individuals with NFG at the first health examination, individuals who shifted to IFG were more, and a shift to DFG after 2 years was associated with much higher risks of stroke and all-cause mortality, compared to persistent NFG. Among individuals with IFG at the first health examination, compared to persistent IFG, individuals who shifted to DFG had higher risks of MI and all-cause mortality.

One study examined the effects of change in fasting glucose over time, which was in line with our results. Fasting glucose variabilities are associated with subsequent risks of MI in non-diabetic patients [19]. Other previous studies were also consistent with our findings, although they did not report a change in fasting glucose but rather one-time fasting glucose level. AusDiab study reported that all-cause mortality was greater for IFG than for NFG among 10,428 participants after a median follow-up period of 5.2 years [11]. Additionally, two meta-analyses showed that elevated fasting glucose was associated with the risk of cardiovascular disease in people without diabetes [12, 13]. A prospective study of a large cohort in Korea reported that IFG is associated with the risk of stroke and coronary heart disease [20]. Another prospective national survey in Israel reported that a linear association between admission blood glucose and 10-year mortality among heart failure patients without diabetes [21].

By contrast, some have reported that IFG is less likely to be a risk factor for cardiovascular disease compared to IGT [7–10]. The differences between the findings of these prior studies and the present study could originate from differences in sample size or from the use of different methods of fasting glucose analysis. Data from several different centers in different countries demonstrate that there is no uniform method for examining fasting glucose [8–10], which can result in the possibility of misclassification of fasting glucose status. However, most single large cohorts such as that mentioned above used a uniform analysis method for evaluating fasting glucose, which led to the indication that an IFG could be a significant predictor for cardiovascular disease risks like our findings.

Compared to persistent IFG, shift to DFG from IFG during 2 years (i.e. more rapid change in fasting glucose compare to shift to persistent IFG) was associated with a higher risk of cardiovascular and all-cause mortality, which could imply that glycemic control could slow or halt the progression of macrovascular complications and all-cause mortality, in line with previous studies [21–24]. In Korea, when participants revealed an IFG status in the national health screening program, they received an advisory opinion via a letter from a doctor, including suggestions for lifestyle modifications and a recommendation for follow-up 3–6 months later in nearby clinics. According to this advisory opinion, people with prediabetes status intentionally aim to elicit a reduction in hyperglycemia through lifestyle modification such as healthy diets, exercise, quitting smoking, or abstemious in drinking. Accordingly, early detection of IFG via screening of glycemic status could be one of the strategy to prevent mortality. However, the shift to NFG from IFG is not significantly associated with the risk of cardiovascular disease, which may be due to minor events.

The Cardiovascular Heart Study in the U.S. reported no evidence that prediabetes is associated with subsequent 13-year incident cardiovascular events or mortality in community-dwelling adults aged more than 65 years [25]. In our subgroup analysis of participants older than 65 years, the shift to IFG or DFG from NFG was not also significantly associated with risk of MI or stroke; however, it was associated with all-cause mortality. Other previous studies were consistent with our results. Among hospitalized patients with heart failure without pre-existing diabetes, there was a linear relationship between admission glucose level and 10-year mortality [21]. Although heart failure could be the direct cause of death, higher fasting glucose may be an additional contributor to mortality. The difference between the present study and Cardiovascular Heart Study in U.S. is the presence of an independent variable (i.e. change in fasting glucose vs. one-time measurement of fasting glucose). Accordingly, although a prediabetic status itself was not significant among the elderly, increasing fasting glucose was associated with all-cause mortality.

There are some possible mechanisms reported. Abnormal glucose status disrupts normal endothelial function by oxidative stress [26], protein kinase C activation, and advanced glycated end product receptor activation [27], thereby accelerates atherosclerotic plaque formation as well as increasing arterial stiffness [28]. IFG status has also been associated with arterial endothelial dysfunction and intima-media thickening [29], which linked to incident MI and stroke [30, 31]. Furthermore, a series of experimental studies demonstrated that variability in blood glucose may be more prejudiced to increase cardiovascular disease than constantly high blood glucose [31–34].

There are limitations to our study that need to be noted. First, the development of MI and stroke confirmed by hospitalization for 2 days or more for the relevant disease based on ICD-10 codes, which are conceivable to underestimate the actual number of cardiovascular disease cases. However, a previous study showed that identifying cardiovascular disease events via the ICD-10 code has an accuracy higher than 80% [35]. Second, future development of diabetes during follow-up period is hard to consider, despite the fact that the risk of major adverse cardiovascular events is significantly greater in diabetic patients who have a longer illness duration [36]. However, we investigated the 2-year change in fasting glucose, which could distinguish the progression to DFG from NFG or IFG status. Third, participants in this study were older than the middle-aged, and the elderly may already have subclinical cardiovascular disease. However, among patients with heart failure, high glucose level was associated with mortality [21]. In addition, some studies reported that target organ damage precedes the clinical appearance of diabetes [37]. Lastly, fasting glucose was assessed through serum, not plasma as was recommended [5], which induced an error in the serum analyses of 1.15% compared to plasma analyses [38]. This error may generate when the sample is stored at room temperature after drawing blood; however, the error is very low because the NHIS recommends refrigeration of the sample at health screening centers. Despite these limitations, these findings show representative results based on data from a nationwide NHIS database [15].

## Additional file

**Additional file 1: Table S1.** Associations between change in fasting glucose from the first health examination and the risk of myocardial infarction, stroke, and all-cause mortality excluding participants whose myocardial infarction, stroke, or death occurred in from January 1, 2006 to December 31, 2006. **Table S2.** Associations between change in fasting glucose at the first health examination and the risk of myocardial infarction, stroke, and all-cause mortality excluding participants whose myocardial infarction, stroke, or death occurred in from January 1, 2006 to December 31, 2007.
