# Supplementary material for: The effect of change in fasting glucose on the risk of myocardial infarction, stroke, and all-cause mortality: a nationwide cohort study
Source: Cardiovasc Diabetol. 2018 Apr 7;17:51. doi: 10.1186/s12933-018-0694-z (PMC5889526; doi:10.1186/s12933-018-0694-z)
Supplement: Supplementary file 1 — Additional file 1: Table S1. Associations between change in fasting glucose from the first health examination and the risk of myocardial infarction, stroke, and all-cause mortality excluding participants whose myocardial infarction, stroke, or death occurred in from January 1, 2006 to December 31, 2006. Table S2. Associations between change in fasting glucose at the first health examination and the risk of myocardial infarction, stroke, and all-cause mortality excluding participants whose myocardial infarction, stroke, or death occurred in from January 1, 2006 to December 31, 2007. [file 12933_2018_694_MOESM1_ESM.docx]

Supplemental Table 1━ Associations between change in fasting glucose from the first health examination and the risk of myocardial infarction, stroke, and all-cause mortality excluding participants whose myocardial infarction, stroke, or death occurred in from January 1, 2006 to December 31, 2006

| Fasting glucose level at baseline (mg/dL) | NFG(<100.0mg/dL) | | | IFG(100.0-125.9mg/dL) | | |
| --- | --- | --- | --- | --- | --- | --- |
| Fasting glucose level after 2-year (mg/dL) | NFG  (<100.0mg/dL) | IFG  (100.0-125.9mg/dL) | DFG  (≥126.0mg/dL) | NFG  (<100.0mg/dL) | IFG  (100.0-125.9mg/dL) | DFG  (≥126.0mg/dL) |
| Risk of MI |  |  |  |  |  |  |
| Subtotal, N | 156,659 | 37,798 | 4,014 | 33,589 | 23,841 | 4,463 |
| Number of cases, n | 619 | 201 | 24 | 171 | 128 | 52 |
| HR | 1 | 1.10 | 1.02 | 1.08 | 1 | 1.79 |
| 95% CI |  | 0.93-1.29 | 0.68-1.54 | 0.86-1.36 |  | 1.29-2.49 |
| Risk of stroke |  |  |  |  |  |  |
| Subtotal, N | 156,301 | 37,700 | 4,001 | 33,478 | 23,766 | 4,443 |
| Number of cases, n | 3,993 | 1,152 | 166 | 1,053 | 794 | 188 |
| HR | 1 | 1.04 | 1.20 | 1.00 | 1 | 1.10 |
| 95% CI |  | 0.98-1.11 | 1.03-1.41 | 0.91-1.10 |  | 0.94-1.29 |
| Risk of all-cause mortality |  |  |  |  |  |  |
| Subtotal, N | 156,301 | 37,681 | 3,993 | 33,472 | 23,747 | 4,434 |
| Number of cases, n | 4,805 | 1,460 | 290 | 1,381 | 1,011 | 259 |
| HR | 1 | 1.08 | 1.58 | 0.98 | 1 | 1.16 |
| 95% CI |  | 1.01-1.14 | 1.40-1.78 | 0.90-1.06 |  | 1.01-1.33 |

Abbreviations: N, n, number; HR, hazard ratio; CI, confidential interval; MI, myocardial infarction.

Hazard ratio analyzed by Cox proportional hazards regression analysis adjusted for age, socioeconomic status, physical activity, smoking status, alcohol consumption, body mass index, blood pressure, total cholesterol, Charlson comorbidity index, and baseline fasting glucose level.

Supplemental Table 2━ Associations between change in fasting glucose at the first health examination and the risk of myocardial infarction, stroke, and all-cause mortality excluding participants whose myocardial infarction, stroke, or death occurred in from January 1, 2006 to December 31, 2007

| Fasting glucose level at baseline (mg/dL) | NFG(<100.0mg/dL) | | | IFG(100.0-125.9mg/dL) | | |
| --- | --- | --- | --- | --- | --- | --- |
| Fasting glucose level after 2-year (mg/dL) | NFG  (<100.0mg/dL) | IFG  (100.0-125.9mg/dL) | DFG  (≥126.0mg/dL) | NFG  (<100.0mg/dL) | IFG  (100.0-125.9mg/dL) | DFG  (≥126.0mg/dL) |
| Risk of MI |  |  |  |  |  |  |
| Subtotal, N | 156,585 | 37,775 | 4,009 | 33,568 | 23,825 | 4,457 |
| Number of cases, n | 545 | 178 | 19 | 150 | 112 | 46 |
| HR | 1 | 1.11 | 0.92 | 1.08 | 1 | 1.82 |
| 95% CI |  | 0.93-1.31 | 0.58-1.46 | 0.84-1.38 |  | 1.29-2.58 |
| Risk of stroke |  |  |  |  |  |  |
| Subtotal, N | 155,843 | 37,562 | 3,979 | 33,361 | 23,666 | 4,430 |
| Number of cases, n | 3,535 | 1,014 | 144 | 936 | 694 | 175 |
| HR | 1 | 1.04 | 1.19 | 1.02 | 1 | 1.18 |
| 95% CI |  | 0.97-1.12 | 1.01-1.40 | 0.92-1.12 |  | 0.99-1.39 |
| Risk of all-cause mortality |  |  |  |  |  |  |
| Subtotal, N | 155,771 | 37,516 | 3,953 | 33,310 | 23,632 | 4,407 |
| Number of cases, n | 4,275 | 1,295 | 250 | 1,219 | 896 | 232 |
| HR | 1 | 1.07 | 1.54 | 0.98 | 1 | 1.17 |
| 95% CI |  | 1.01-1.14 | 1.36-1.75 | 0.89-1.07 |  | 1.02-1.36 |

Abbreviations: N, n, number; HR, hazard ratio; CI, confidential interval; MI, myocardial infarction.

Hazard ratio analyzed by Cox proportional hazards regression analysis adjusted for age, socioeconomic status, physical activity, smoking status, alcohol consumption, body mass index, blood pressure, total cholesterol, Charlson comorbidity index, and baseline fasting glucose level.
